# Supplementary material for: Detection of Antimicrobial Resistance, Pathogenicity, and Virulence Potentials of Non-Typhoidal Salmonella Isolates at the Yaounde Abattoir Using Whole-Genome Sequencing Technique
Source: Pathogens. 2022 Apr 23;11(5):502. doi: 10.3390/pathogens11050502 (PMC9148033; doi:10.3390/pathogens11050502)
Supplement: Supplementary file 1 [file pathogens-11-00502-s001.zip › pathogens-1627350-supplementary.pdf]

Table S1. Summary of genome analysis of non-typhoidal *Salmonella* isolated from the Yaoundé abattoir, Cameroon

GenBank accession no.	No. coding genes	No. RNA	No. ncRNAs	No. tRNAs	No. CRISPR arrays	No. contigs	Total length (Mb)	GC%	Subspecies	Predicted serotype	MLST
PYKK00000000	5,336	85	12	63	2	211	4,611,182	52	*enterica*	Poona	ST-308
PYKJ00000000	4,799	98	15	71	2	50	4,762,368	52	*enterica*	Enteritidis	ST-11
PYKI00000000	5,881	89	13	63	3	359	4,536,152	52	*enterica*	Poona	ST-308
PYKH00000000	4,630	98	11	66	2	138	4,769,090	52	*enterica*	Wilhelmsburg	ST-3311
PYKG00000000	4,641	105	12	73	2	183	4,772,851	52	*enterica*	Wilhelmsburg	ST-3311
PYKF00000000	4,886	96	12	64	2	84	4,785,147	52	*enterica*	Wilhelmsburg	ST-3311
PYKE00000000	4,587	83	12	63	3	347	4,707,411	51	*enterica*	Wernigerode	ST-4585
PYKD00000000	4,655	105	14	72	2	230	4,715,897	52	*enterica*	Poona	ST-308
PYKC00000000	4,608	78	11	62	2	173	4,746,203	52	*enterica*	Wilhelmsburg	ST-3311
PYKB00000000	5,270	68	9	48	2	175	4,771,862	52	*enterica*	Wilhelmsburg	S-3311
PYKA00000000	4,561	84	13	63	2	141	4,718,495	52	*enterica*	Wilhelmsburg	S-3311
PYJZ00000000	4,624	84	13	64	2	142	4,756,935	51.4	*enterica*	Wilhelmsburg	S-3311
PYJY00000000	4,775	96	13	70	2	332	4,818,121	52	*enterica*	Enteritidis	ST-11
PYJX00000000	4,562	99	14	66	2	251	4,645,907	52	*enterica*	Infantis	ST-1823
PYJW00000000	4,507	99	15	64	3	194	4,634,563	51	*enterica*	Infantis	ST-1824
PYJV00000000	4,605	83	13	64	2	132	4,751,175	51	*enterica*	Wernigerode	ST-4585
PYJU00000000	4,646	107	12	71	2	92	4,794,148	52	*enterica*	Wernigerode	ST-4584
PYJT00000000	4,622	88	14	66	2	300	4,734,751	51	*enterica*	Wilhelmsburg	S-3311
PYJR00000000	4,550	82	12	62	2	142	4,672451	51.2	*enterica*	Kibusi	ST-4584

Table S2. Antimicrobial sensitivity of non-typhoidal *Salmonella* isolated at the Yaounde abattoir

Sample code	*Salmonella* isolate	Tetracycline (Ø= mm)	Chloramphenicol (Ø= mm)	Streptomycin (Ø= mm)	Ampicillin (Ø= mm)
34ev	Wernigerode	30	34	24	22
8ev	Poona	9 (≤ 11)	7 (≤ 12)	6 (≤ 11)	6 (≤ 13)
35dea	Wilhelmsburg	28	35	25	22
35deb	Wilhelmsburg	30	31	24	20
22sa	Poona	10 (≤ 11)	6 (≤ 12)	6 (≤ 11)	6 (≤ 13)
31eva	Wilhelmsburg	33	40	25	22
31evb	Wilhelmsburg	38	46	25	20
32eva	Wilhelmsburg	35	40	25	25
32evb	Wernigerode	30	31	25	23
86ev	Infantis	27	35	8	14 (14-16)
100ev	Wernigerode	32	40	24	22
36ev	Wilhelmsburg	29	38	25	22
98se	Wernigerode	32	40	26	18
108ev	Kibusi	30	38	25	17
20de	Enteritidis	32	35	8 (≤ 11)	22
34de	Poona	10 (≤ 11)	10 (≤ 12)	6(≤ 11)	7 (≤ 13)
60sa	Enteritidis	34	35	13 (12-14)	29
88sa	Infantis	32	40	25	24
88sab	Infantis	33	38	24	22
133sa	Enteritidis	32	36	25	14 (14-16)
103bo	Wilhelmsburg	34	40	24	20
EVJUL	Mbandaka	28	33	9 (≤ 11)	18
DEF1	Not sequenced	30	36	22	20

NB: Zone of inhibition in red indicates resistance to the test antibiotic; blue = intermediate; in black are the numbers representing the diameter of zone of inhibition mean susceptible to the test antibiotics; code and serovar of *Salmonella* strain written in red, represent the multidrug-resistant (MDR) strains.

Table S3. Function of each virulence factor (See ResFinder 1.0)

Category	Function
1	Iron uptake system, sit operon
2	P4-like integrase ; located within a high-pathogenicity-island (HPI) region
3	Type VI secretion system effector
4	Multi drug resistance (ampicillin, gentamicin, streptomycin, spectinomycin, sulfathiazole, tetracycline, and nalidixic acid)
5	Type III secretion system, invasion into epithelial cells, apoptosis (InvA, OrgA, SptP, SipA, SipB, SipC, SipD, SopE, prgH)
6	Phage 46 and the sefA-R chaperone-usher fimbrial operon
7	sopB, mppA, icdA, envF, msgA, envE, pagD, pagC
8	msgA, narP
9	Acetyl-coA dehydrolase (gacD)
10	LysR family transcriptional regulation (gtrB)
11	Trancriptional regulation (gtrA)
12	Electron transfer favoprotein beta subunit (gpiA)
13	Trancriptional regulation (gpiB)
14	Type III secretion system, required for systemic infection and intracellular pathogenesis by facilitating replication of intracellular bacteria within membrane-bound *Salmonella*-containing vacuoles
15	Invasion, survival in monocytes, Mg2+ uptake (MgtC, B, MarT, MisL)
16	Invasion, survival in monocytes, Mg2+ uptake (sugR, rhuMMgtC, B, MarT, MisL), putative fimbrial-like protein(yadC/K./L/M), probable pilin chaperone(ecpD1/D2)
17	Type I secretion system, putative toxin secretion, apoptosis, required for intracellular survival in macrophages; large secretion protein(siiE) and Type I secretion system components(siiC,D,F), genes weakly similar to RTX-like toxins
18	Effector proteins for SPI-1 and SPI-2 (SopB, SigD, PipB)
19	safA-D and tcsA-R chaperone-usher fimbrialoperons6
20	Vi exopolysaccharide, SopE prophage and a type IVB pilus operon
21	Two bacteriocin pseudogenes, genes conferring immunity to the bacteriocins
22	Type I secretory apparatus, large RTX-like protein
23	Intestinal colonization and persistence determinants (shdA, ratA, ratB, sivI, sivH)

Table S4. *Salmonella* pathogenicity island-encoded effector proteins

Isolates	Protein effector	Function	Origin
8ev, 20de, 31eva, 31evb,32eva, 32evb 34de, 34ev, 35dea, 35deb, 36ev, 60sa, 88sa, 88sab, 98se, 100ev, 103bo, 108ev	Cell adherence/invasion proteins : SipA, EnvF, InvA, InvE, SipB, SipC, SipD, SopB, SopE, Sii E, IagB, SptP, protein oxygen-regulated invasion protein OrgB, PhoP, PhoH protein, protein MisL, host colonization factor (ShdA), protein rtn T 6SS protein IcmF	involved in adherence, cell aggregation, biofilm formation, cell invasion, resistance to antimicrobial peptides, internalization and survival within macrophages	*S*. Enteritidis P125109, *S*. Paratyphi A ATCC 9150, *S*. Newport SL254, *S*. Agona SL483, *S*. Typhimurium LT2, *S*. Schwarzengrund *S*. Choleraesuis SC-B67, *S*. Heidelberg SL476
31eva, 31evb, 32eva, 34de, 34ev, 36ev,88sab, 98se, 100ev, 103bo	OmpL	Outer membrane protein with high immunogenicity potential	*S*. Schwarzengrund, *S*. Newport SL254
20de, 22sa, 31evb, 32evb, 36ev, 60sa, 88sab	Heat shock protein, Stress proteins	Small polypeptides that play important role during *Salmonella* pathogenesis	*S*. Choleraesuis SC-B67, *Klebsiella pneumoniae*
8ev, 20de, 31eva, 31evb, 32eva, 34de, 34ev, 35dea, 36ev, 60sa, 88sa, 88sab, 98se, 100ev, 103bo, 108ev	T3SS effector protein SopA, SopE, secreted effector protein SopE2, virulence proteins SopD and SopD2, Effector proteins pipB2, SpiC, SpvB, SseB, SseC, and SseD, T6SS lysozyme-related protein, Ais protein, effector proteins IpaD/SipD, protein bax	Promote *Salmonella* survival replication within macrophages, critical for virulence in different hosts, in invasion of epithelial cells and intestinal inflammation, translocation of SPI-1, SPI2 effector proteins<br><br>Involved in proapoptotic activity	*S*. Enteritidis P125109, *S*. Paratyphi A ATCC 9150, *S*. Newport SL254, *S*. Paratyphi C RKS4594 *S*. Schwarzengrund, *S*. Agona SL483<br>*S*. Heidelberg SL476
20de,31eva,31evb, 32eva, 34de,34ev, 36ev, 60sa,88sa, 88sab, 98se, 100ev,108ev	Antigen presentation protein SpaK, SpaN, SpaS, SpaR,	Responsible for the surface presentation of determinants needed for the entry of *Salmonella* species into host cells	*S*. Agona SL483, Typhimurium LT2, *S*. Newport SL254r. SL483
8ev, 20de, 31eva, 31evb, 32eva, 32evb, 34de, 34ev, 36ev, 60sa, 88sa, 88sab, 98se, 103bo, 108ev	EsrB, secretion system apparatus SsaV, SsaH and SsaM, SPI-6 encoded Rhs- protein	Response regulators of secreted effectors and host inflammatory responses	*S*. Schwarzengrund CVM19633, *S*. Newport SL254

Table S5a. Distribution of adhesion factors, toxins and virulence plasmids

Isolate	Virulence determinant	Function	Origin
8ev,20de, 31eva, 31evb, 32eva, 34de, 34ev,35dea,36ev,60sa,88sab,98se, 100ev, 103bo,108ev	Transcriptional regulator CsgD	Master transcriptional regulator in biofilm formation	*S.* Schwarzengrund CVM19633
20de, 31eva, 31evb, 32eva, 34de, 34ev,35dea, 36ev, 60sa, 88sa, 88sab, 98se, 100ev, 103bo, 108ev	Flagellin: Protein FliZ, FliT	Class 2 flagellar genes acting as repressor genes	*S.* Paratyphi A ATCC 9150, *S.* Newport SL254
20de,31eva,31evb,32eva,34de,34ev, 36ev,60sa,88sa,88sab,98se,100ev, 103bo,108ev	Pilin chaperon ecpD1	encoded on SPI-3 and expressed with increasing temperature above 22°C	*S.* Choleraesuis SC-B67, *S.* Agona SL483
8ev, 20de, 31evb, 32eva, 34de,60sa,88sa,88sab, 108ev	Putative entericidin A	Bacterial antidote/toxin peptides for maintain plasmids stability	*S.* Typhimurium. LT2, *S.* Paratyphi C RKS4594
20de, 31evb,32eva, 34ev, 36ev, 88sa, 88sab, 98se, 100ev, 108ev	Flavodoxin	Small soluble electron transfer protein acting as bactericidal molecules	*S.* Schwarzengrund
20de,22sa,31eva, 31evb,35dea,32eva,34de, 34ev, 36ev, 60sa, 88sa, 88sab, 98se, 100ev, 103bo, 108ev	Thioredoxin 1 (TrxA)	Protects *Salmonella* against the oxidative stress	*Escherichia coli* 536
8ev, 31eva, 31evb, 32eva, 32evb, 34ev, 35dea, 36ev, 88sa, 88sab, 98se, 100ev, 103bo	Putative fimbriae; usher: FimA, FimC, FimZ, HilD,	Play key role in adhesion of *Salmonella* to the host cell receptors	*S.* Heidelberg SL476, *S.* Enteritidis P125109. Agona. SL483, *S.* Newport SL254
22sa, 31eva, 31evb, 32evb, 35dea, 36ev, 103bo	Transposase for element IS200	Transposons playing key role in host gene expression control	*S.* Paratyphi A ATCC 9150
20de, 60sa,88sab, 98se, 100ev	Putative ABC-type bacteriocin exporter	Export of bacteriocin and autoprotection	*S.* Newport str. SL254 *S.* Typhimurium LT2
60sa, 100ev	Putative shiga-like toxin A subunit	E. coli derived toxin	*E. coli* 536

Table S5b. Distribution of adhesion factors, and virulence plasmid (continued)

Isolates	Virulence determinant	Function	Origin
8ev, 22sa, 34de	Col (PHAD28) plasmid	Harbor gene *qnrB* encoding resistance against fluoroquinolone	*S*. Hadar HAD28
8ev, 20de,34de, 60sa	IncFII (S) and Incl1-1 (α) plasmids	Harbor toxin SpvB and mediate resistance against aminoglycosides, tetracycline, chloramphenicol, sulfonamides, β-lactams, fluoroquinolones	*S*. Paratyphi C RKS4594<br>*S*. Typhimurium
34de	IncH12 and IncH12A plasmids	Mediate resistance to aminoglycosides, tetracycline, chloramphenicol, sulfonamides, β-lactams, fluoroquinolones	*Serratia mrescens* K478
8ev, 20de, 34de 31eva, 32eva, 32evb, 35dea, 36ev, 88sab, 98se, 100ev	Proteins CuSA & ScsC	Regulate the intracellular excess of copper and promotes intramacrophage survival	*S*. Agona SL483, *S*. Typhimurium LT2<br>*S*. Newport SL254
8ev,20de,31eva,31evb,32evb, 34de,34ev,35dea,36ev,60sa,88sa,98se,100ev,103bo,108ev 88sab	Putative Lys R family	Regulates many cellular processes (activators and repressors, N-terminal DNA binding domain)	*S*. Paratyphi A ATCC9150, *S*. Typhimurium LT2
34de, 60sa, 88sa, 88sab, 98se, 100ev	Virulence genes ter C, tehA, Terx, Terw	Plasmid-borne genes coding for tellerium ion protein resistance	*E. coli* plasmid pJIE186-2
34de	cvaC and mchF genes	Microcin V genes for the synthesis of microcin	*E. coli* plasmid^{PCOLVCA7V}
